# Supplementary material for: A Likelihood Approach for Real-Time Calibration of Stochastic Compartmental Epidemic Models
Source: PLoS Comput Biol. 2017 Jan 17;13(1):e1005257. doi: 10.1371/journal.pcbi.1005257 (PMC5240920; doi:10.1371/journal.pcbi.1005257)
Supplement: S1 File — (TAR.GZ) [file pcbi.1005257.s014.tar.gz › HSPH_Online-SI-Revision/Additions/IPoi10/n100-extreme-SEITR/n100-extreme-SEITR_BENCH2_table8.pdf]

1	newly	3 weeks	spec	108.	816.676	{373., 1422.}	656.182	0.821151	newly	3 weeks	cumul	716.	3345.59	{1611., 5632.}	367.261	0.615911	newly	5 weeks	spec	52.	459.549	{227., 750.}	783.749	0.889586	newly	5 weeks	cumul	854.	4414.37	{2124., 7434.}	416.905	0.659143	newly	43 weeks	cumul	939.	5752.27	{2799., 9732.}	512.595	0.732298
2	newly	3 weeks	spec	102.	414.077	{216., 760.}	305.958	0.541825	newly	3 weeks	cumul	593.	2068.68	{1070., 3668.}	248.849	0.476878	newly	5 weeks	spec	36.	168.938	{80., 337.}	369.274	0.600737	newly	5 weeks	cumul	673.	2504.17	{1265., 4503.}	272.091	0.50489	newly	50 weeks	cumul	712.	2809.22	{1409., 5047.}	294.553	0.530014
3	newly	3 weeks	spec	169.	113.217	{39., 201.}	52.2978	0.330975	newly	3 weeks	cumul	920.	654.783	{238., 1059.}	50.9366	0.303947	newly	5 weeks	spec	52.	37.7667	{13., 66.}	51.859	0.310343	newly	5 weeks	cumul	1072.	758.683	{277., 1232.}	51.1769	0.306214	newly	52 weeks	cumul	1137.	808.5	{298., 1314.}	51.1815	0.30464
4	newly	3 weeks	spec	137.	272.631	{136., 411.}	102.055	0.253218	newly	3 weeks	cumul	790.	1349.69	{687., 2181.}	76.4051	0.203498	newly	5 weeks	spec	41.	111.431	{51., 166.}	171.782	0.37147	newly	5 weeks	cumul	909.	1635.4	{829., 2617.}	84.133	0.218791	newly	50 weeks	cumul	985.	1832.14	{898., 2965.}	89.5869	0.229882
5	newly	3 weeks	spec	34.	87.1538	{38., 138.}	157.511	0.321206	newly	3 weeks	cumul	171.	531.4	{214., 840.}	210.76	0.400192	newly	5 weeks	spec	11.	26.6615	{10., 44.}	147.413	0.306034	newly	5 weeks	cumul	195.	606.154	{259., 956.}	210.848	0.400771	newly	52 weeks	cumul	201.	639.231	{270., 1016.}	218.025	0.410789
6	newly	3 weeks	spec	5.	95.7077	{41., 165.}	1814.15	1.20679	newly	3 weeks	cumul	60.	543.292	{259., 940.}	805.487	0.885479	newly	5 weeks	spec	2.	30.	{15., 53.}	1400.	1.10897	newly	5 weeks	cumul	63.	626.462	{305., 1092.}	894.383	0.927252	newly	51 weeks	cumul	64.	663.077	{321., 1164.}	936.058	0.944635
7	newly	3 weeks	spec	469.	952.375	{380., 1680.}	113.806	0.225967	newly	3 weeks	cumul	2142.	3602.66	{1545., 6738.}	84.5428	0.205434	newly	5 weeks	spec	221.	615.031	{199., 902.}	182.82	0.269038	newly	5 weeks	cumul	2717.	4981.92	{1991., 8857.}	97.8394	0.213727	newly	43 weeks	cumul	3226.	7050.84	{2640., 11 591.}	128.265	0.231451
8	newly	3 weeks	spec	225.	467.618	{224., 922.}	109.804	0.246005	newly	3 weeks	cumul	1088.	2325.26	{1162., 4130.}	114.606	0.258853	newly	5 weeks	spec	80.	194.309	{93., 357.}	143.033	0.304321	newly	5 weeks	cumul	1321.	2822.13	{1372., 5031.}	114.52	0.257778	newly	50 weeks	cumul	1472.	3162.94	{1541., 5622.}	115.721	0.259755
9	newly	3 weeks	spec	19.	80.4722	{42., 137.}	323.977	0.581607	newly	3 weeks	cumul	167.	495.097	{258., 854.}	197.264	0.429697	newly	5 weeks	spec	5.	24.9028	{12., 44.}	398.056	0.646436	newly	5 weeks	cumul	179.	564.931	{288., 958.}	216.209	0.456322	newly	52 weeks	cumul	188.	594.264	{300., 1012.}	216.63	0.457101
10	newly	3 weeks	spec	613.	419.732	{206., 733.}	41.6133	0.250103	newly	3 weeks	cumul	2661.	1902.39	{959., 3285.}	39.9689	0.233506	newly	5 weeks	spec	260.	198.036	{90., 351.}	38.7775	0.217473	newly	5 weeks	cumul	3324.	2390.86	{1185., 4122.}	39.8573	0.231834	newly	48 weeks	cumul	3949.	2836.18	{1399., 4851.}	39.8519	0.232143
11	newly	3 weeks	spec	448.	504.121	{297., 767.}	32.4202	0.126928	newly	3 weeks	cumul	2137.	2234.97	{1331., 3369.}	28.9914	0.121712	newly	5 weeks	spec	237.	245.833	{136., 373.}	30.2327	0.129883	newly	5 weeks	cumul	2689.	2832.47	{1672., 4249.}	29.4426	0.122821	newly	47 weeks	cumul	3221.	3400.52	{2038., 5068.}	29.6906	0.12381
12	newly	3 weeks	spec	209.	684.141	{288., 1323.}	227.785	0.426945	newly	3 weeks	cumul	1086.	3191.65	{1260., 6006.}	194.85	0.386007	newly	5 weeks	spec	68.	301.746	{121., 521.}	343.745	0.554928	newly	5 weeks	cumul	1294.	3954.11	{1561., 7391.}	206.394	0.4014	newly	48 weeks	cumul	1442.	4569.14	{1804., 8518.}	217.485	0.414734
13	newly	3 weeks	spec	300.	319.797	{186., 458.}	35.4948	0.137705	newly	3 weeks	cumul	1508.	1524.73	{893., 2264.}	34.0548	0.139417	newly	5 weeks	spec	131.	138.953	{78., 216.}	35.8182	0.140518	newly	5 weeks	cumul	1818.	1872.83	{1091., 2785.}	34.369	0.137813	newly	49 weeks	cumul	2109.	2139.22	{1264., 3177.}	34.5039	0.140733
14	newly	3 weeks	spec	681.	995.69	{397., 1615.}	65.6967	0.200324	newly	3 weeks	cumul	2981.	3967.07	{1675., 6306.}	57.137	0.190146	newly	5 weeks	spec	331.	578.662	{226., 919.}	87.5537	0.233485	newly	5 weeks	cumul	3718.	5306.39	{2230., 8387.}	63.3826	0.198131	newly	42 weeks	cumul	4474.	7132.56	{2938., 11 355.}	74.7228	0.212798
15	newly	3 weeks	spec	238.	704.733	{321., 1096.}	196.106	0.403928	newly	3 weeks	cumul	1233.	2888.48	{1391., 4336.}	135.111	0.309789	newly	5 weeks	spec	100.	395.733	{200., 599.}	295.733	0.528813	newly	5 weeks	cumul	1479.	3816.5	{1841., 5784.}	158.159	0.34769	newly	43 weeks	cumul	1667.	4983.42	{2361., 7607.}	198.945	0.409815
16	newly	3 weeks	spec	233.	597.864	{293., 1118.}	157.179	0.331475	newly	3 weeks	cumul	1269.	2541.68	{1239., 4888.}	103.106	0.236663	newly	5 weeks	spec	91.	304.242	{141., 591.}	234.332	0.43873	newly	5 weeks	cumul	1497.	3270.38	{1561., 6322.}	119.8	0.266058	newly	46 weeks	cumul	1647.	4045.29	{1931., 7823.}	146.081	0.31234
17	newly	3 weeks	spec	1.	194.154	{85., 351.}	19 315.4	2.20802	newly	3 weeks	cumul	62.	1103.78	{510., 2056.}	1680.3	1.16962	newly	5 weeks	spec	0.1	66.1692	{30., 124.}	66 069.2	2.73902	newly	5 weeks	cumul	64.	1281.49	{597., 2387.}	1902.33	1.22089	newly	52 weeks	cumul	64.	1367.34	{644., 2533.}	2036.47	1.24872
18	newly	3 weeks	spec	93.	274.169	{106., 503.}	195.765	0.382547	newly	3 weeks	cumul	605.	1504.69	{602., 2875.}	151.212	0.318748	newly	5 weeks	spec	53.	96.9538	{34., 173.}	99.7678	0.250997	newly	5 weeks	cumul	722.	1763.71	{691., 3365.}	147.311	0.313049	newly	51 weeks	cumul	795.	1902.25	{750., 3587.}	142.748	0.306112
19	newly	3 weeks	spec	465.	1144.84	{554., 2061.}	147.266	0.331106	newly	3 weeks	cumul	2284.	4860.98	{2271., 8815.}	115.101	0.276853	newly	5 weeks	spec	217.	596.459	{270., 1081.}	175.44	0.372269	newly	5 weeks	cumul	2812.	6284.69	{2948., 11 407.}	125.061	0.293382	newly	46 weeks	cumul	3336.	7802.56	{3676., 14 283.}	135.046	0.31
20	newly	3 weeks	spec	75.	197.274	{95., 371.}	163.29	0.36124	newly	3 weeks	cumul	401.	1060.76	{540., 1971.}	164.657	0.36242	newly	5 weeks	spec	14.	68.7581	{35., 121.}	391.129	0.634261	newly	5 weeks	cumul	459.	1244.9	{631., 2308.}	171.221	0.373203	newly	51 weeks	cumul	486.	1347.5	{677., 2521.}	177.263	0.382346
21	newly	3 weeks	spec	38.	168.625	{88., 290.}	343.75	0.595715	newly	3 weeks	cumul	270.	983.781	{509., 1715.}	264.363	0.510241	newly	5 weeks	spec	12.	56.1563	{29., 99.}	367.969	0.614442	newly	5 weeks	cumul	303.	1138.34	{593., 1986.}	275.691	0.523418	newly	52 weeks	cumul	316.	1214.86	{629., 2122.}	284.449	0.53326
22	newly	3 weeks	spec	162.	261.389	{101., 516.}	79.904	0.22519	newly	3 weeks	cumul	806.	1234.83	{514., 2337.}	73.1079	0.211976	newly	5 weeks	spec	58.	116.847	{49., 244.}	110.704	0.270039	newly	5 weeks	cumul	951.	1528.46	{637., 2916.}	77.7909	0.216992	newly	48 weeks	cumul	1042.	1759.39	{744., 3381.}	83.9145	0.225563
23	newly	3 weeks	spec	22.	291.333	{152., 503.}	1224.24	1.06533	newly	3 weeks	cumul	142.	1464.59	{786., 2606.}	931.4	0.957797	newly	5 weeks	spec	3.	121.873	{66., 226.}	3962.43	1.55198	newly	5 weeks	cumul	156.	1773.67	{947., 3170.}	1036.97	1.00014	newly	49 weeks	cumul	161.	1987.92	{1055., 3533.}	1134.73	1.03559
24	newly	3 weeks	spec	51.	248.033	{101., 504.}	386.34	0.603673	newly	3 weeks	cumul	312.	1345.17	{555., 2862.}	331.143	0.551341	newly	5 weeks	spec	15.	88.8167	{33., 168.}	492.111	0.68038	newly	5 weeks	cumul	360.	1580.13	{652., 3322.}	338.926	0.55913	newly	51 weeks	cumul	369.	1708.72	{722., 3549.}	363.067	0.582382
25	newly	3 weeks	spec	160.	788.508	{491., 1113.}	392.817	0.66539	newly	3 weeks	cumul	871.	3504.37	{2169., 5008.}	302.339	0.577311	newly	5 weeks	spec	60.	391.415	{231., 591.}	552.359	0.784985	newly	5 weeks	cumul	1024.	4447.09	{2773., 6402.}	334.286	0.610206	newly	47 weeks	cumul	1086.	5358.62	{3341., 7681.}	393.427	0.665459
26	newly	3 weeks	spec	96.	888.984	{387., 1759.}	826.025	0.874216	newly	3 weeks	cumul	682.	3769.63	{1663., 7607.}	452.732	0.652858	newly	5 weeks	spec	33.	468.127	{193., 955.}	1318.57	1.05358	newly	5 weeks	cumul	784.	4881.73	{2127., 9832.}	522.67	0.702868	newly	45 weeks	cumul	878.	6039.3	{2626., 12 217.}	587.848	0.744575
27	newly	3 weeks	spec	572.	493.136	{233., 811.}	46.0188	0.219416	newly	3 weeks	cumul	2438.	2095.3	{992., 3266.}	45.5067	0.217929	newly	5 weeks	spec	284.	262.015	{115., 413.}	48.0741	0.21666	newly	5 weeks	cumul	3101.	2717.21	{1273., 4254.}	45.951	0.216215	newly	45 weeks	cumul	3756.	3379.97	{1571., 5324.}	46.2799	0.212374
28	newly	3 weeks	spec	8.	47.5152	{20., 65.}	493.939	0.674663	newly	3 weeks	cumul	54.	292.621	{138., 390.}	441.891	0.645079	newly	5 weeks	spec	1.	15.5758	{6., 24.}	1457.58	1.0819	newly	5 weeks	cumul	61.	335.636	{159., 451.}	450.224	0.652026	newly	52 weeks	cumul	63.	353.242	{168., 474.}	460.702	0.659248
29	newly	3 weeks	spec	378.	227.017	{98., 357.}	58.8845	0.361098	newly	3 weeks	cumul	1806.	1084.9	{483., 1663.}	58.0971	0.357391	newly	5 weeks	spec	145.	96.85	{41., 149.}	55.046	0.318053	newly	5 weeks	cumul	2196.	1330.65	{611., 2020.}	57.8635	0.353596	newly	49 weeks	cumul	2473.	1514.53	{694., 2297.}	57.8124	0.351159
30	newly	3 weeks	spec	146.	192.319	{84., 360.}	58.1298	0.174349	newly	3 weeks	cumul	831.	1116.07	{488., 1969.}	60.4405	0.178734	newly	5 weeks	spec	47.	64.6377	{27., 123.}	66.3275	0.196807	newly	5 weeks	cumul	975.	1289.8	{568., 2254.}	59.7414	0.179473	newly	52 weeks	cumul	1051.	1375.26	{606., 2393.}	59.3334	0.180644
31	newly	3 weeks	spec	292.	876.213	{434., 1318.}	200.073	0.418479	newly	3 weeks	cumul	1423.	3517.07	{1746., 5272.}	147.159	0.336526	newly	5 weeks	spec	119.	506.393	{248., 773.}	325.541	0.567968	newly	5 weeks	cumul	1719.	4688.05	{2289., 6992.}	172.72	0.378336	newly	42 weeks	cumul	1955.	6276.69	{3054., 9373.}	221.058	0.448271
32	newly	3 weeks	spec	532.	397.155	{177., 674.}	46.3281	0.253934	newly	3 weeks	cumul	2380.	1866.72	{847., 3332.}	45.058	0.238226	newly	5 weeks	spec	254.	173.741	{79., 282.}	47.8075	0.282781	newly	5 weeks	cumul	3005.	2307.72	{1048., 4147.}	45.3612	0.243926	newly	48 weeks	cumul	3596.	2659.91	{1232., 4734.}	46.1859	0.255378
33	newly	3 weeks	spec	65.	24.8065	{9., 40.}	66.9975	0.53874	newly	3 weeks	cumul	390.	149.839	{67., 269.}	67.2787	0.525789	newly	5 weeks	spec	28.	7.48387	{2., 15.}	74.8848	0.743104	newly	5 weeks	cumul	468.	170.726	{74., 308.}	68.3175	0.547319	newly	52 weeks	cumul	508.	180.613	{78., 322.}	68.9421	0.558377
34	newly	3 weeks	spec	107.	347.115	{175., 515.}	224.406	0.452317	newly	3 weeks	cumul	548.	1633.44	{821., 2503.}	198.073	0.417024	newly	5 weeks	spec	35.	154.803	{75., 231.}	342.295	0.585824	newly	5 weeks	cumul	648.	2023.21	{997., 3129.}	212.224	0.43697	newly	48 weeks	cumul	692.	2354.11	{1149., 3636.}	240.19	0.474162
35	newly	3 weeks	spec	10.	88.3929	{36., 123.}	783.929	0.84975	newly	3 weeks	cumul	81.	528.446	{264., 766.}	552.403	0.725917	newly	5 weeks	spec	0.1	26.4107	{12., 38.}	26 310.7	2.31847	newly	5 weeks	cumul	82.	603.321	{296., 875.}	635.758	0.777585	newly	52 weeks	cumul	82.	633.875	{309., 923.}	673.018	0.798785
36	newly	3 weeks	spec	587.	961.729	{459., 1798.}	72.7355	0.19715	newly	3 weeks	cumul	2638.	4311.99	{2045., 8041.}	71.741	0.195769	newly	5 weeks	spec	275.	460.071	{199., 854.}	77.5429	0.209035	newly	5 weeks	cumul	3306.	5438.73	{2538., 10 169.}	72.9535	0.198375	newly	48 weeks	cumul	3908.	6469.1	{2978., 12 142.}	73.9702	0.199956
37	newly	3 weeks	spec	138.	446.176	{237., 782.}	223.316	0.464936	newly	3 weeks	cumul	721.	2125.51	{1072., 3566.}	194.801	0.425445	newly	5 weeks	spec	53.	195.206	{96., 331.}	268.313	0.517512	newly	5 weeks	cumul	854.	2613.25	{1347., 4346.}	206.001	0.441272	newly	49 weeks	cumul	922.	2988.99	{1514., 4986.}	224.185	0.466302
38	newly	3 weeks	spec	103.	658.116	{211., 1290.}	538.948	0.682766	newly	3 weeks	cumul	598.	2889.72	{1008., 5733.}	383.232	0.568277	newly	5 weeks	spec	29.	329.377	{113., 627.}	1035.78	0.921845	newly	5 weeks	cumul	693.	3681.32	{1295., 7295.}	431.215	0.606821	newly	46 weeks	cumul	752.	4444.	{1551., 8777.}	490.957	0.651182
39	newly	3 weeks	spec	188.	479.613	{194., 878.}	156.057	0.334578	newly	3 weeks	cumul	946.	2248.	{960., 4191.}	139.416	0.31263	newly	5 weeks	spec	61.	216.419	{87., 436.}	254.786	0.475753	newly	5 weeks	cumul	1112.	2784.95	{1181., 5211.}	151.472	0.331478	newly	48 weeks	cumul	1224.	3214.02	{1340., 5960.}	162.989	0.34927
40	newly	3 weeks	spec	395.	230.522	{126., 392.}	47.9501	0.301502	newly	3 weeks	cumul	1868.	1121.24	{628., 1896.}	47.0796	0.291408	newly	5 weeks	spec	218.	103.746	{58., 176.}	55.6415	0.381675	newly	5 weeks	cumul	2391.	1381.13	{786., 2354.}	48.4597	0.305684	newly	49 weeks	cumul	2892.	1576.73	{887., 2689.}	50.53	0.32716
41	newly	3 weeks	spec	12.	194.304	{81., 324.}	1519.2	1.09533	newly	3 weeks	cumul	121.	1106.04	{471., 1855.}	814.086	0.847034	newly	5 weeks	spec	2.	63.9855	{25., 97.}	3099.28	1.38244	newly	5 weeks	cumul	135.	1280.23	{527., 2121.}	848.32	0.862669	newly	52 weeks	cumul	137.	1364.59	{562., 2260.}	896.054	0.883407
42	newly	3 weeks	spec	675.	975.308	{503., 1622.}	58.0194	0.166946	newly	3 weeks	cumul	2966.	3910.88	{2049., 6583.}	49.9678	0.155547	newly	5 weeks	spec	340.	562.185	{287., 961.}	72.6697	0.189355	newly	5 weeks	cumul	3794.	5212.98	{2709., 8754.}	52.9046	0.158115	newly	43 weeks	cumul	4690.	6945.37	{3579., 11 573.}	59.5608	0.167701
43	newly	3 weeks	spec	545.	493.443	{170., 902.}	63.9817	0.298137	newly	3 weeks	cumul	2315.	2219.8	{763., 3977.}	63.1496	0.277801	newly	5 weeks	spec	245.	238.2	{86., 415.}	65.4694	0.28185	newly	5 weeks	cumul	2918.	2806.34	{973., 4946.}	63.6179	0.278608	newly	48 weeks	cumul	3370.	3335.54	{1159., 5863.}	64.2442	0.273078
44	newly	3 weeks	spec	215.	608.973	{268., 1086.}	183.243	0.371363	newly	3 weeks	cumul	1211.	2837.23	{1304., 5023.}	134.978	0.296245	newly	5 weeks	spec	93.	273.068	{121., 508.}	194.005	0.38517	newly	5 weeks	cumul	1475.	3515.64	{1620., 6228.}	138.902	0.302416	newly	48 weeks	cumul	1690.	4059.36	{1875., 7246.}	140.776	0.305364
45	newly	3 weeks	spec	201.	453.658	{204., 764.}	126.885	0.277125	newly	3 weeks	cumul	1070.	2259.35	{1069., 3663.}	113.175	0.255515	newly	5 weeks	spec	90.	185.253	{81., 306.}	109.803	0.248548	newly	5 weeks	cumul	1275.	2738.91	{1291., 4461.}	116.608	0.261528	newly	50 weeks	cumul	1419.	3076.51	{1433., 5021.}	118.555	0.264699
46	newly	3 weeks	spec	138.	46.6935	{25., 77.}	66.3978	0.521976	newly	3 weeks	cumul	722.	274.597	{154., 419.}	62.2531	0.457913	newly	5 weeks	spec	50.	15.1129	{8., 24.}	69.7742	0.566578	newly	5 weeks	cumul	861.	316.694	{178., 480.}	63.4015	0.471776	newly	52 weeks	cumul	942.	337.71	{191., 513.}	64.249	0.482581
47	newly	3 weeks	spec	224.	414.051	{214., 724.}	88.0221	0.222832	newly	3 weeks	cumul	1089.	1863.68	{1020., 3291.}	76.3941	0.199139	newly	5 weeks	spec	77.	196.169	{98., 349.}	156.042	0.348274	newly	5 weeks	cumul	1298.	2346.41	{1272., 4166.}	84.448	0.214829	newly	47 weeks	cumul	1446.	2785.2	{1484., 4949.}	95.397	0.236898
48	newly	3 weeks	spec	86.	397.177	{139., 595.}	361.834	0.502237	newly	3 weeks	cumul	493.	1968.29	{702., 3034.}	299.248	0.446392	newly	5 weeks	spec	18.	163.435	{59., 258.}	807.975	0.800135	newly	5 weeks	cumul	561.	2386.35	{852., 3663.}	325.375	0.472942	newly	50 weeks	cumul	581.	2679.16	{975., 4037.}	361.129	0.50718
49	newly	3 weeks	spec	117.	172.072	{76., 294.}	65.2546	0.188952	newly	3 weeks	cumul	638.	816.29	{379., 1387.}	54.7045	0.179707	newly	5 weeks	spec	40.	74.3478	{33., 124.}	94.7826	0.231844	newly	5 weeks	cumul	753.	1003.83	{465., 1684.}	57.3224	0.180751	newly	48 weeks	cumul	828.	1146.87	{537., 1900.}	59.8824	0.182049
50	newly	3 weeks	spec	261.	332.618	{176., 559.}	46.2925	0.159081	newly	3 weeks	cumul	1311.	1573.84	{775., 2758.}	43.9326	0.160044	newly	5 weeks	spec	104.	141.662	{68., 257.}	55.1895	0.182907	newly	5 weeks	cumul	1579.	1935.56	{938., 3441.}	44.9465	0.160377	newly	49 weeks	cumul	1754.	2208.01	{1085., 3987.}	46.4308	0.161471
